# Epistasis among *Drosophila persimilis* Factors Conferring Hybrid Male Sterility with *D. pseudoobscura bogotana*

**DOI:** 10.1371/journal.pone.0015377

**Published:** 2010-10-27

**Authors:** Audrey S. Chang, Sarah M. Bennett, Mohamed A. F. Noor

**Affiliations:** 1 Biology Department, Duke University, Durham, North Carolina, United States of America; 2 Department of Biology, New York University, New York, New York, United States of America; University of Texas Arlington, United States of America

## Abstract

The Bateson-Dobzhansky-Muller model posits that hybrid incompatibilities result from genetic changes that accumulate during population divergence. Indeed, much effort in recent years has been devoted to identifying genes associated with hybrid incompatibilities, often with limited success, suggesting that hybrid sterility and inviability are frequently caused by complex interactions between multiple loci and not by single or a small number of gene pairs. Our previous study showed that the nature of epistasis between sterility-conferring QTL in the *Drosophila persimilis*-*D. pseudoobscura bogotana* species pair is highly specific. Here, we further dissect one of the three QTL underlying hybrid male sterility between these species and provide evidence for multiple factors *within* this QTL. This result indicates that the number of loci thought to contribute to hybrid dysfunction may have been underestimated, and we discuss how linkage and complex epistasis may be characteristic of the genetics of hybrid incompatibilities. We further pinpoint the location of one locus that confers hybrid male sterility when homozygous, dubbed “*mule-like*”, to roughly 250 kilobases.

## Introduction

Hybrid dysfunction (e.g., sterility and inviability) in animals evolves in part as a pleiotropic by-product of genic divergence when populations become isolated and subsequently accumulate changes as a result of selection or genetic drift. The Bateson-Dobzhansky-Muller (BDM) model [1], [2], [3] describes how, when these genetically differentiated populations hybridize, genes that evolved in one genomic context fail to function in the background of the other genome, leading to decreased fitness of the hybrid offspring. Much effort has been directed toward identifying genes that contribute to hybrid incompatibilities and other forms of reproductive isolation.

Elucidation of the identity and molecular function of incompatibility genes may eventually reveal the types of changes that lead to reproductive isolation. However, understanding the nature of the evolution of reproductive isolation also requires answers to other long-standing and fundamental questions: How many loci are necessary and sufficient to produce hybrid dysfunction? For example, can an allele at a single locus cause sterility when placed in a foreign genetic background (i.e., are there “single factors of large effect”)? Is epistasis between foreign, introgressed alleles important? What is the linkage relationship between loci causing hybrid problems?

Studies in Drosophila and other systems have provided some early insights: hybrid male sterility (HMS) between some species results from complex epistasis between alleles at multiple loci; single factors of large effect on HMS as considered in the basic BDM model appear rare or absent [4], [5], [6], [7], [8], [9], [10], [11] but see [12]. A recent study of yeast spore failure in hybrids further suggests that postzygotic isolation may also rely on complex interactions between multiple loci [13], but other studies of incompatibility in yeast hybrids have not detected such complexity [14]. In some instances, introgression of multiple linked factors is necessary to produce infertile hybrid males [11], [15]. However, we are far from being able to generalize the number of loci, or the pattern of epistasis between them, that underlies hybrid sterility.

Mapping factors that cause hybrid dysfunction often employs methodology similar to those for mapping quantitative traits, but because hybrid sterility is studied as a binary (or categorical) and threshold trait, two distinctions are important. First, in most studies, individuals are classified as “sterile” or “fertile” (or sometimes with 3–4 categories based on sperm or offspring number), such that quantitative reductions in fertility are generally not measured. Second, once an individual is sterile, further “sterility” effects cannot be observed. These problems become especially acute because specific alleles at several loci may interact to cause sterility and these interactions can vary in complexity. For instance, multiple independent sterility-conferring interactions may exist: alleles at loci A and B can interact to cause sterility, and a separate interaction between loci C and D may also be sufficient. Alternatively, a single interaction involving alleles at A, B, C, and D may be necessary. Many standard backcross mapping studies do not distinguish between these scenarios.

To overcome the limitations of backcross analyses, much progress in identifying and counting hybrid sterility loci has thus come from introgression analyses [5], [7], [8], [11], [16], [17], [18], [19]. Such analyses allow the evaluation of the contributions of individual or groups of genetic factors, as well as the interactions between them. Alleles from one species that are introgressed into another species background necessarily interact with the native alleles present. However, the foreign (i.e., introgressed) factors may also interact with each other: we [20] previously showed that some foreign factors can be introgressed with no detectable reduction in fertility, but exhibit strong sterility effects when in combination, even when all are heterozygous. Introgression analyses searching for hybrid-sterility-conferring alleles often ignore this type of epistasis.

This study builds on the results from our previous studies and further dissects two individual QTL that confer hybrid male sterility when introgressed from *Drosophila persimilis* into a *D. pseudoobscura bogotana* genetic background. These two species diverged approximately 0.5 to 1 million years ago [21], [22] and are distinguished by four chromosomal inversions: two on the X chromsome, one on the 2^nd^-chromosome, and one on the 3^rd^-chromosome. The QTL examined in this study resides outside the inverted region on chromosome-2 [23].

In this study, we show that, between *D. persimilis* and *D. p. bogotana*, what appeared to be a single QTL in fact had 2–3 tightly linked loci conferring sterility. Hence, epistasis between more loci than could be estimated from a standard QTL mapping design confers infertility of backcross hybrid males. Furthermore, we observed that at least one of these loci is “sufficient” to confer complete sterility if made homozygous in a foreign background.

## Materials and Methods

### Fly stocks and culture conditions

*Drosophila pseudoobscura bogotana* carrying a *white* eye mutation were used in the crosses described below, as the *white* locus is linked to an inversion that distinguishes between *D. p. bogotana* and *D. persimilis*. The *D. p. bogotana white* strain is a subculture of the *D. p. bogotana* El Recreo line collected in 1978 (provided by H. A. Orr). *The D. persimilis* MSH1993 line was derived from females collected at Mt. St. Helena, California in 1993 [24]. All crosses were performed on standard sugar/yeast/agar medium at 20±1°C and 85% relative humidity.

### Fine-mapping of sterility factors within the chromosome-2 QTL

**Figure 1:**
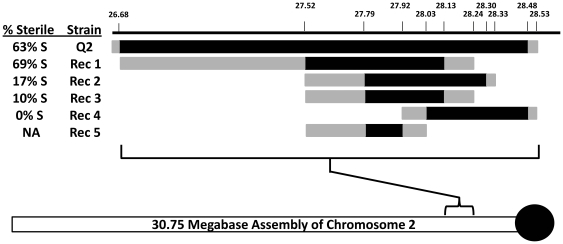
Fraction of males that were sterile when heterozygous for the specified *D. persimilis* chromosome 2 segment (black bars) as well as *D. persimilis* QTL onp, chromosomes 3 and 4. Grey bars indicate areas of uncertainty of origin because they lie between genotyped markers. The positions noted are based on the complete *D. pseudoobscura* genome sequence assembly [26] of chromosome 2 (see bottom of figure, black circle indicating centromere), and are presented in megabases (e.g., 27.92 indicates assembly position 27,920,000). Fertility for Rec5 is not presented because it was assayed in homozygous form and without the QTL on chromosomes 3 and 4.

Following the methods described in Chang and Noor [20], introgression lines were created to break up the previously described chromosome-2 QTL (hereafter Q2). Briefly, interspecies F_1_ females were backcrossed to *D. p. bogotana* males for ten generations to purge the background of *D. persimilis* alleles at regions other than those coinciding with the focal Q2 QTL. During this process, the *D. p. bogotana* chromosomal arrangements were selected for the two inversions on the X-chromosome and the one inversion on the 2^nd^-chromosome that differentiate the two species. This ensures that the mapping results obtained here recapture effects detected in the original QTL-mapping study [23]. Selection for the foreign introgressed Q2 segment and for the rearrangements was completed by microsatellite genotyping of markers delineating the QTL and the rearrangements. The final line bore only the Q2 segment from *D. persimilis* in a *D. p. bogotana* genetic background (see Figure 1). Marker sequences are available in Table S1.

For each introgressed *D. persimilis* QTL region, we sought to generate recombinant segments and assay their fertility alone and in combination with the other QTL. Additional mapping lines were created by repeatedly crossing females of the Q2 introgression line to *D. p. bogotana* males. Recombination between the *D. persimilis* and *D. p. bogotana* genomes within each QTL generated multiple independent lines; additional microsatellite markers were designed to differentiate between these lines. Because we previously identified an interaction between the QTL on each of the three major autosomes as necessary for complete sterility [20], [23], females from mapping lines were then crossed to males heterozygous for the chromosome-3 and either the chromosome-4 or the chromosome-2 QTL. Male flies *heterozygous* for one portion of the Q2 QTL (Recombinant lines 1–4: see Figure 1) and *heterozygous* for the other two QTL (Q3 and Q4) were assayed for fertility following the methods of [25].

Finally, we assayed the *homozygous* fertility effect of one of the smallest of the individual introgressed Q2 recombinants by crossing males and females from the line and assaying sperm motility in the homozygous offspring (not bearing *D. persimilis* alleles at Q3 or Q4). We further backcrossed this recombinant line to *D. p. bogotana* to generate a smaller recombinant line, and this newer line was also assayed for fertility as above.

## Results

### Fine-mapping dominant chromosome-2 factors underlying hybrid male sterility

Using introgressions generated by recombination between the *D. persimilis* and *D. p. bogotana* genomes (see Materials and Methods), we assessed the effect on hybrid male sterility of four regions within the chromosome-2 QTL (recombinants 1–4 in Figure 1: hereafter referred to as Rec 1, Rec 2, etc.), which spans almost 2 Mb near the centromere. A *D. persimilis* allele of each of these regions was introgressed into a *D. p. bogotana* background along with a copy of the *D. persimilis* alleles of Q3 and Q4, as sterility is manifest only upon co-introgression of all three QTL [20]. We first assessed the male fertility effects of the recombinant segments in heterozygous state using flies also heterozygous Q3 and Q4.

Introgression of a *D. persimilis* segment of approximately 1.5 Mb (Rec 1 in figure 1) resulted in high (∼70%) hybrid male sterility. However, introgression of an overlapping segment between 0.5 Mb and 0.8 Mb in size (Rec 2 in figure 1) dramatically decreased the proportion of sterile males to approximately 17%, suggesting that at least one sterility factor resides between positions 26,680,000 and 27,790,000 of chromosome 2. This factor is necessary for causing near-complete sterility, as its absence had a significant effect on the proportion of sterile males (compare Rec 1 to Rec 2 and Rec 3). Furthermore, two smaller but overlapping introgressions that span the length of recombinant 2 exhibited ∼10% male sterility in Rec 3 and no sterility of all males with recombinant 4. The difference in sterility between Rec 2 and 3 is suggestive albeit not statistically significant, suggesting the presence of at least two and potentially three sterility-conferring loci within the overall Q2 region. At this smaller genomic scale (within a single autosomal QTL), we still observe multiple factors contributing to the resultant hybrid male sterility.

### A single *D. persimilis* locus conferring complete hybrid male sterility when homozygous in a *D. p. bogotana* genetic background

Our previous work [20] demonstrated that dominance of hybrid male sterility alleles can be increased by interactions with heterozygous alleles at loci on other chromosomes. Further, some regions showing heterozygous hybrid sterility effects in combination with other alleles are “individually sufficient” to cause complete hybrid sterility if made *homozygous*. To potentially localize such a sufficient sterility-conferring allele with higher resolution, we examined one of the smallest of our recombinant regions, recombinant 3 (hereafter, Rec3), for its homozygous fertility effect. Like the full Q2 region [20], Rec3 also produces complete hybrid male sterility when homozygous, even in the absence of the *D. persimilis* Q3 and Q4 alleles. However, males heterozygous for Rec3 but lacking the *D. persimilis* Q3 and Q4 alleles are fully fertile. Hence, Rec3 is recessive in its sterility effect when introgressed alone yet can produce complete hybrid male sterility when homozygous.

We generated a small recombinant within Rec3 to attempt to pinpoint one hybrid-sterility-conferring allele's location (designated Rec5). We attempted to generate many more recombinants in this region, and while we identified at least 3 independent ones, they all spanned exactly the same Rec5 window and are considered here together. In contrast to Rec3, males homozygous for Rec5 were fully fertile. Hence, at least one factor causing homozygous male sterility lies between the right end of Rec5 and the right end of Rec3 in Figure 1. By further defining the ends of the Rec3 and Rec5 introgressions, we infer that a *D. persimilis* hybrid male sterility allele resides between *D. pseudoobscura* assembly [26] positions 27.95 Mb and 28.21 Mb: a region of under 250 kb. We name this factor “*mule-like*.”

## Discussion

In our previous study [20], we showed that hybrid male sterility between *Drosophila persimilis* and *D. pseudoobscura bogotana* results from highly specific epistasis involving three autosomal QTL alleles derived from *D. persimilis* as well as an unknown number of alleles from *D. p. bogotana*. Epistasis involving multiple loci leading to hybrid sterility has been documented before [5], [6], [7], [11], [15], [27]. However, because few studies have dissected the individual QTL contributing to hybrid sterility, the minimum number of genetic factors that underlie this form of intrinsic postzygotic isolation remains unclear.

By fine-mapping the sterility loci residing within one of these QTL, we show that the sterility phenotype in this hybridization results from interactions involving more foreign genetic factors than previously inferred (a minimum of 4) and that some of these factors are tightly linked to one another. Further, these four *D. persimilis* alleles all contribute to this phenotype along with an unknown number of *D. p. bogotana* alleles to cause hybrid male sterility. Finally, one of these four *D. persimilis* alleles rests within an inverted region on chromosome 3 and could therefore easily have multiple factors contributing to this same interaction. Hence, we have just begun to understand the complexity associated with hybrid male sterility, in this system or in general.

### Epistasis among foreign alleles at multiple loci causes hybrid male sterility

To date, only a small number of studies have pinpointed genes that underlie hybrid male sterility between closely related species. Most of the examples that exist are restricted to the genus Drosophila [17], [28], [29], [30] but see [31]. The first gene identified, *OdsH*, causes a 50% reduction in fertility in hybrid males when the *Drosophila mauritiana* allele is introgressed into a *D. simulans* genetic background [15]. For complete sterility, *OdsH* must be co-introgressed with other closely linked factors; these factors remain unidentified. More recently, Phadnis and Orr [29] identified *Overdrive*, an X-linked gene that causes both hybrid male sterility and meiotic drive between the two subspecies (USA and Bogota) of *D. pseudoobscura*. However, this gene must interact with at least two other unidentified X-linked loci from the Bogota subspecies for significant sterility/drive to occur [6]. Thus, between these two young subspecies, introgression of multiple heterospecific loci appears necessary for hybrid male sterility, just as we observed for the *D. p. bogotana* and *D. persimilis* hybridization.

However, cointrogression of foreign alleles does not appear to be universally necessary for hybrid male sterility. Using the *D. simulans* and *D. mauritiana*, Tao et al. [17] identified one region on the 3^rd^ chromosome (*tmy*) capable of causing significant sterility when introgressed on its own.

Beyond just the numbers of alleles contributing to hybrid sterility, some researchers have suggested that hybrid male sterility often may be caused by alleles at closely linked loci. For example, *OdsH* itself had minimal effect on fertility unless a proximal region on the X-chromosome of *D. mauritiana* was co-introgressed with *OdsH* into an otherwise *D. simulans* genetic background [15], [32]. In the *D. pseudoobscura* species group, the importance of linkage between heterospecific alleles in causing hybrid male sterility is unclear and may vary across hybridizations. In the more recent divergence between the two subspecies of *D. pseudoobscura*, close linkage was not observed: *Overdrive* interacts with loci from *D. p. bogotana* on a different chromosome arm to cause sterility in hybrids [6], [29]. However, between the more distantly related species *D. persimilis* and *D. p. bogotana*, multiple closely linked factors (i.e., within the same QTL region) are needed to cause sterility in hybrids at least some of the time. (Note that the linked factors studied here must interact also with each other and with the factors on other chromosomes for hybrid male sterility to occur.)

Together, these studies and ours here reveal a fundamental gap in our knowledge of the genetics underlying postzygotic barriers to gene flow. While the identities, and thus the function, of these incompatibility genes are becoming available, the nature and number of interactions among genes that confer these hybrid dysfunctions remains unknown. Knowledge of the nature of interactions may provide additional clues to the molecular underpinnings and evolution of hybrid dysfunction. Addressing this deficiency is far from trivial, as it requires simultaneous introgression of, and generating recombinants of, multiple sterility-conferring QTL regions. The resolution limitations imposed by the simple mapping techniques generally employed places us just at the tip of the iceberg.

### A fine-mapped sterility-conferring allele from *D. persimilis*

We have pinpointed the location of a *D. persimilis* allele conferring hybrid male sterility when *homozygous* in a *D. p. bogotana* genetic background. This region (Rec3 in Figure 1) extends just over 500 kilobases. We further identified a recombinant of that region (Rec5) that does not produce sterility when homozygous. We can infer that at least one locus necessary for homozygous hybrid male sterility (“*mule-like*”) resides in the region covered by Rec3 but not Rec5- a region of approximately 250 kilobases. Given the small size, this could be a single locus “sufficient” to cause complete hybrid male sterility in a foreign background, but we cannot rule out that one or more factors within Rec5 are also required. Further, we cannot rule out until this factor is isolated that it arose by spontaneous mutation and is not found in naturally occurring *D. persimilis*. We are continuing to dissect the Rec3 window to define the exact position of *mule-like*.

We emphasize that we still know nothing of the interactions involved in causing hybrid male sterility. The *D. persimilis* allele at *mule-like* may interact with a great many alleles at *D. p. bogotana* loci. Further, while we infer *mule-like* to be a single gene, our results showing extensive interactions among closely linked loci leading to sterility may apply to homozygous introgressions as well. As with much research in speciation genetics, each step forward in data collection is accompanied by a much smaller step forward in understanding the underlying complexity.

## Supporting Information

Table S1 Raw data from sterility assays and microsatellite primer sequences used for genotyping.
(XLS)
